# Allogeneic Cardiospheres Delivered via Percutaneous Transendocardial Injection Increase Viable Myocardium, Decrease Scar Size, and Attenuate Cardiac Dilatation in Porcine Ischemic Cardiomyopathy

**DOI:** 10.1371/journal.pone.0113805

**Published:** 2014-12-02

**Authors:** Kristine Yee, Konstantinos Malliaras, Hideaki Kanazawa, Eleni Tseliou, Ke Cheng, Daniel J. Luthringer, Chak-Sum Ho, Kentaro Takayama, Naoto Minamino, James F. Dawkins, Supurna Chowdhury, Doan Trang Duong, Jeffrey Seinfeld, Ryan C. Middleton, Rohan Dharmakumar, Debiao Li, Linda Marbán, Raj R. Makkar, Eduardo Marbán

**Affiliations:** 1 Cedars-Sinai Heart Institute, Los Angeles, California, United States of America; 2 Department of Molecular Biomedical Sciences and Center for Comparative Medicine and Translational Research, College of Veterinary Medicine, North Carolina State University, Raleigh, North Carolina, United States of America; 3 Joint Department of Biomedical Engineering, University of North Carolina at Chapel Hill and North Carolina State University, North Carolina, United States of America; 4 Gift of Life Michigan, Ann Arbor, Michigan, United States of America; 5 National Cerebral and Cardiovascular Center Research Institute, Osaka, Japan; 6 Cedars-Sinai Biomedical Imaging Research Institute, Los Angeles, California, United States of America; 7 Capricor, Beverly Hills, California, United States of America; Georgia Regents University, United States of America

## Abstract

**Background:**

Epicardial injection of heart-derived cell products is safe and effective post-myocardial infarction (MI), but clinically-translatable transendocardial injection has never been evaluated. We sought to assess the feasibility, safety and efficacy of percutaneous transendocardial injection of heart-derived cells in porcine chronic ischemic cardiomyopathy.

**Methods and Results:**

We studied a total of 89 minipigs; 63 completed the specified protocols. After NOGA-guided transendocardial injection, we quantified engraftment of escalating doses of allogeneic cardiospheres or cardiosphere-derived cells in minipigs (n = 22) post-MI. Next, a dose-ranging, blinded, randomized, placebo-controlled (“dose optimization”) study of transendocardial injection of the better-engrafting product was performed in infarcted minipigs (n = 16). Finally, the superior product and dose (150 million cardiospheres) were tested in a blinded, randomized, placebo-controlled (“pivotal”) study (n = 22). Contrast-enhanced cardiac MRI revealed that all cardiosphere doses preserved systolic function and attenuated remodeling. The maximum feasible dose (150 million cells) was most effective in reducing scar size, increasing viable myocardium and improving ejection fraction. In the pivotal study, eight weeks post-injection, histopathology demonstrated no excess inflammation, and no myocyte hypertrophy, in treated minipigs versus controls. No alloreactive donor-specific antibodies developed over time. MRI showed reduced scar size, increased viable mass, and attenuation of cardiac dilatation with no effect on ejection fraction in the treated group compared to placebo.

**Conclusions:**

Dose-optimized injection of allogeneic cardiospheres is safe, decreases scar size, increases viable myocardium, and attenuates cardiac dilatation in porcine chronic ischemic cardiomyopathy. The decreases in scar size, mirrored by increases in viable myocardium, are consistent with therapeutic regeneration.

## Introduction

Approximately six million Americans [1] and 23 million people worldwide [2] suffer from chronic heart failure. While traditional therapies aim at attenuating disease progression, regenerative cell therapy seeks to reverse heart failure by regrowing healthy working myocardium. To that end, numerous cell types and delivery methods have been studied [3], [4], [5]. Cells derived from the heart are particularly attractive; intracoronary infusion of autologous cardiosphere-derived cells (CDCs) [6], [7] or c-kit+ heart-derived cells [8] has shown promising results in patients with post-ischemic ventricular dysfunction (although doubt has been cast regarding data presented in SCIPIO, the c-kit+ cell clinical study) [9]. While intracoronary delivery is safe and convenient, cell retention is low [10]. Since increased cellular retention has been associated with greater long-term benefits on cardiac function both in animal models [11], [12], [13] and in humans [14], [15], there is good reason to believe that development of delivery methods with better engraftment might enhance the efficacy of cell therapy, especially in the setting of chronic ischemic cardiomyopathy, where local homing signals are reduced [16]. Preclinical and clinical studies have shown that intramyocardial (IM) cell delivery leads to greater cardiac engraftment compared to intracoronary delivery [15], [17], [18], [19], [20], [21], although agreement on this point is not universal [22], [23], [24]. Intramyocardial injection also enables the use of cardiospheres (CSps, three-dimensional spherical clusters of heart-derived cells), which are more efficacious than CDCs when both human products are delivered IM in immunodeficient mice with acute MI [25]. In pigs, open-chest epicardial injection of CDCs and CSps is safe and effective post-MI [26]. However, clinically-translatable transendocardial injection of heart-derived cell products has never been reported.

We initially sought to optimize dosing and delivery protocols for transendocardial injection of heart-derived cell products, and then to test the optimized process in chronic porcine ischemic cardiomyopathy. First, we compared 24-hour engraftment of allogeneic CSps and CDCs using NOGA-guided transendocardial injection. Second, we performed a dose-ranging study of the better-engrafting cell product. Finally, we performed a pivotal, randomized, blinded, placebo-controlled study to investigate if dose-optimized transendocardial injection of heart-derived cells is safe and effective in a porcine model of chronic ischemic cardiomyopathy. Our aims were successfully achieved and this method has proved to be safe and effective.

## Methods

All animal studies were performed in an American Association for Accreditation of Laboratory Animal Care accredited facility with approval from the Institutional Animal Care and Use Committee of the Cedars-Sinai Medical Center. All procedures were performed using full analgesia and anesthesia, when appropriate, and all efforts were made to eliminate suffering.

**Figure 1:**
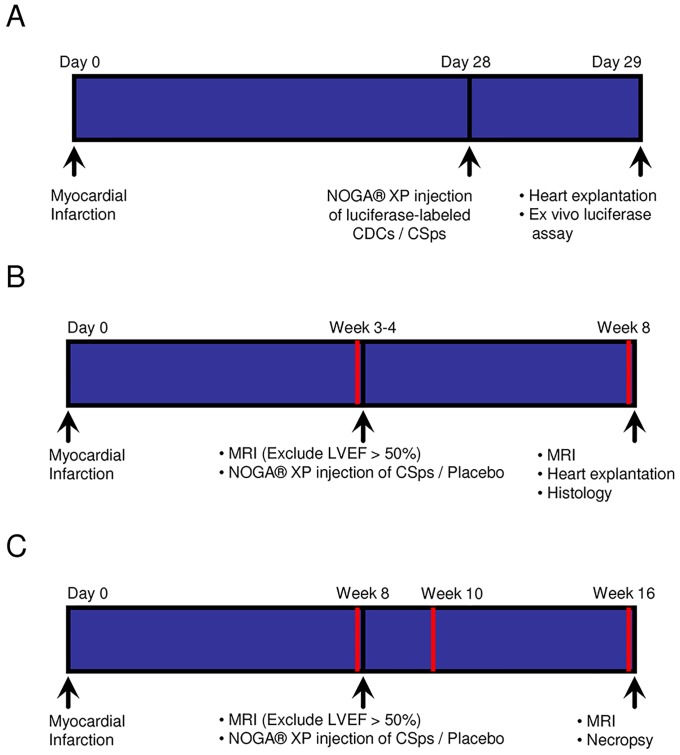
Schematic depiction of the timeline for three experimental protocols. A) Engraftment Study. B) Dose Optimization Study. C) Pivotal Study. Red lines indicate timepoints of blood draws.

Three separate experimental protocols were performed, as depicted schematically in Figure 1. A total of 89 pigs were studied: 22 completed the engraftment study (Figure 1A); 16 completed the dose optimization study (Figure 1B); 22 completed the pivotal study (Figure 1C); 26 were excluded per protocol for procedural mortality or high ejection fraction; and 3 were used for allosensitization protocols. Three pigs served as heart donors for derivation of cell products.

### Engraftment study

#### Cell culture

A freshly-explanted heart from one male farm pig was minced into multiple ∼500 µm^3^ pieces and digested with collagenase IV (Sigma Aldrich, St. Louis, MO). These explants were plated onto fibronectin (BD Biosciences, San Jose, CA)-coated dishes with cardiac explant medium (IMDM [Invitrogen, Carlsbad, CA], 20% FBS, 1% penicillin-streptomycin, 1% L-glutamine, 0.1 mM 2-mercaptoethanol) and expanded at 37°C for two weeks. The outgrowth of adherent cells was harvested and re-plated into poly-D-lysine (BD, Franklin Lakes, NJ)-coated wells. Within three days, the majority of the cells gave rise to three-dimensional, free-floating clusters of cells termed primary CSps. Free-floating primary CSps were collected and plated onto fibronectin-coated dishes. The primary CSps adhered and flattened to form a monolayer of CDCs [7], [27], which were transduced with an adenovirus expressing the firefly luciferase gene. For animals receiving luciferase-labeled CDCs, cells were harvested on the day of injection and injected at this stage. For animals receiving luciferase-labeled CSps, CDCs were re-plated into poly-D-lysine-coated wells to form secondary CSps. Secondary CSps were harvested and filtered immediately prior to injection with a 160 µm nylon net filter (Millipore, Billerica, MA). The maximum feasible dose of CSps was determined to be 100–200 million isolated cell equivalents (ICEs) delivered as CSps (see Results).

#### Induction of MI and transendocardial cell injection

Thirty-three adult Yucatan minipigs (15±5 months) weighing 52±9 kg were studied. A porcine model of post-MI ischemic cardiomyopathy was used [28], [29]. On day 0, animals were premedicated with ketamine 20 mg/kg IM, atropine 0.05 mg/kg IM, and acepromazine 0.25 mg/kg IM. They were induced with propofol 2–4 mg/kg IV to effect, intubated, and maintained on isoflurane 2–3%. Amiodarone (10 mg/kg IV loading dose, then 0.5 mg/kg IV PRN) and lidocaine (0.03 µg/kg/min CRI) were given to prevent/treat ventricular arrhythmias. Heparin 100 IU/kg IV was given for anticoagulation. A coronary dilation catheter (Trek OTW 2–3 mm, Abbott Vascular, Santa Clara, CA) was inflated in the mid-LAD (immediately after the 1^st^ diagonal branch), providing 100% occlusion for 2.5 hours. Post-operative oxymorphone 0.15 mg/kg IM BID PRN and carprofen 4 mg/kg PO q24h PRN were administered for analgesia.

**Figure 2:**
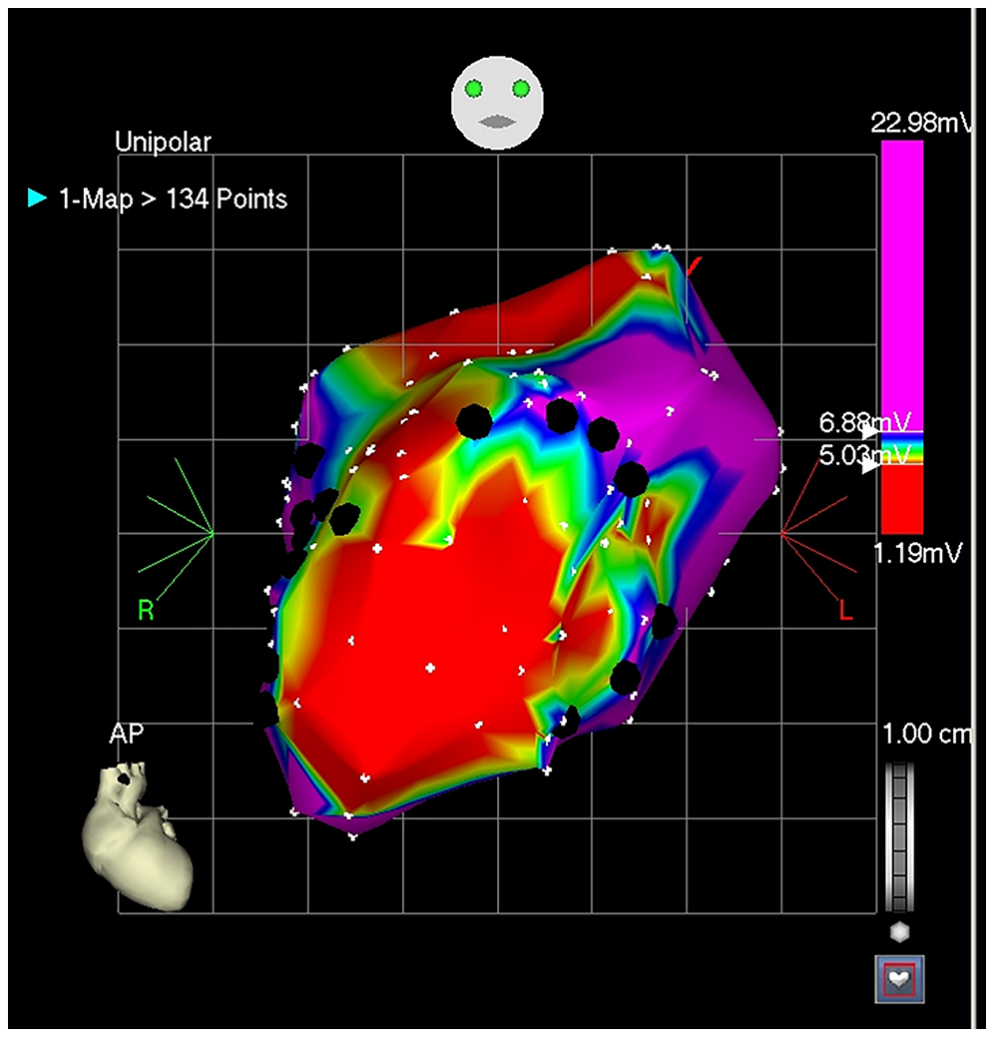
NOGA-guided transendocardial injections. Left ventricular electroanatomic map created by the NOGA XP Cardiac Navigation System. Red indicates scar tissue. Black dots indicate injection points into the infarct border zone.

On day 28, animals were re-anesthetized using the same protocol and drug regimen. Pigs were randomized to receive luciferase-labeled cells: a) high-dose CSps (10 million ICEs); b) low-dose CSps (5 million ICEs); c) high-dose CDCs (10 million CDCs); d) low-dose CDCs (5 million CDCs); and e) maximum feasible dose CSps (100–200 million ICEs). Randomization for all arms of the study was performed by the cell manufacturing laboratory staff, with the remaining staff blinded throughout experimentation and primary data analysis. An electroanatomical map of the left ventricle (LV) was created using the NOGA XP Cardiac Navigation System (Biosense Webster, Inc., Diamond Bar, CA). Cells were injected by a single investigator blinded to infusate identity into the LV peri-infarct region (border zone) (Figure 2) after verification of loop stability <3 mm and the presence of a ventricular premature contraction immediately upon needle extrusion. Border zone was defined by a unipolar voltage of 6.9–10 mV upon endocardial apposition of the catheter tip. Total cell dosage was divided into 10 to 15 injection sites, of ∼150 µl per injection. Twenty-four hours later, the animal was humanely euthanized and the heart, lung, liver, spleen, and kidney were excised.

#### Ex vivo luciferase assay

Luciferase assay (depicted schematically in Supporting Image S1A) was performed as described [30]. This method is advantageous because only viable cells can generate a signal since the reaction requires transcription, translation, and ATP, though possible false decreases in signal can occur due to gene silencing [31]. Cells were transduced with an adenoviral vector carrying the firefly luciferase gene three days prior to injection. For each animal, a separate standard curve was constructed. Specifically, once the cell dose was allocated for injection, an aliquot of cells (from the same batch of adenovirally-transduced cells used for that specific pig) was cultured for an additional 24 hours to create the animal-specific standard curve. Twenty four hours after cell delivery, the injected regions of the LV were homogenized and analyzed for luminescence. We chose to analyze the totality of the injected tissue (rather than isolated myocardial samples) to minimize sampling error and accurately measure cardiac engraftment; however, this approach prevented us from investigating the localization of the retained cells (within the injected region) by immunohistochemistry. Animal-specific standard curves were prepared by measuring the luminescence from known numbers of transduced cells (Supporting Image S1B), with conversion to a cell number by reference to the respective standard curve. Thus, transduction efficiency is equal in the standard curve and in the cell products delivered, so that results for each particular animal are not confounded. Twenty-four hour engraftment was expressed as a percentage of cells originally injected. Off-target engraftment of cells in lung, spleen, liver, and kidney was similarly quantified. This validated method has high sensitivity and reproducibility [26].

### Dose Optimization Study

#### Cell Culture

A freshly-explanted heart from one male farm pig was cultured as above, with the luciferase labeling step omitted, to generate secondary CSps; these were harvested and filtered immediately prior to injection with a 160 µm nylon net to maintain a uniform maximum size.

#### Induction of MI and transendocardial cell injection

Twenty four adult Yucatan minipigs (9±2 months; 2 male, 22 female) weighing 44±5 kg were studied. On day 0, animals underwent induction of MI by 100% balloon occlusion of the mid-LAD for 2.5 hours, as described above. In the 4^th^ week post-MI, pigs were then re-anesthetized and received, by random assignment: a) vehicle (PBS); b) low-dose CSps (15 million ICEs); c) high-dose CSps (45 million ICEs), or; d) maximum feasible dose CSps (150 million ICEs). An electroanatomical map of the LV was created and cells were injected as described above (Figure 2) by a single investigator blinded to infusate identity. Vehicle/cells were divided into 15 injection sites of ∼150 µl per injection. On week 8, animals were re-anesthetized for euthanasia and heart explantation.

#### Cardiac MRI

To assess efficacy, baseline (just before intervention in the 4^th^ week) and endpoint (week 8) contrast-enhanced cardiac MRI was performed and analyzed by two investigators blinded to treatment allocation, as described [30], [32], using the same anesthetic protocol described above. Contrast-enhanced MRI has been shown to accurately measure scarred and viable myocardium after cell therapy and therefore represents an excellent tool for monitoring regenerative efficacy [32]. Animals with LV ejection fraction (LVEF)>50% at baseline were excluded from the protocol and replaced (n = 4). This cutoff was chosen because normal LVEF in minipigs is reported to be 54±1.7% [33], indicating that minimal injury had occurred if LVEF remained>50%. LVEF drops to 45.6±10.4% one month post-MI in the Yucatan minipig when infarct size is 10% or greater [34]. Total LV mass, scar mass, scar size (scar mass÷LV mass), viable myocardial mass (LV mass minus scar mass), end-diastolic volume (EDV), end-systolic volume (ESV), LVEF, and indices of regional function (end-systolic wall thickness, wall thickening, wall motion) were measured.

#### Histocompatibility

Low-resolution swine leukocyte antigen typing was performed on the donor farm pig and all recipient minipigs as described [35], [36] by a single investigator blinded to treatment allocation. Divergent pig strains were used to simulate fully allogeneic conditions in human transplantation where donor and recipient are mismatched with regard to human leukocyte antigens (HLA).

#### Circulating anti-donor alloreactive antibodies

To evaluate humoral immune response, recipient pig serum was obtained at time points t = 4 weeks (baseline) and 8 weeks (4 weeks post-injection). Serum was screened with flow cytometry for circulating anti-donor IgG antibodies at both time points. An allosensitization protocol was performed to provide controls for comparison as described [37], [38]. Peripheral bone marrow mononuclear cells (PBMMNCs) were harvested from a donor farm pig at four time points (t = 0, 7, 14, 21 days). Serum from the donor pig was tested for circulating anti-donor antibodies at t = 0; this served as the negative control. PBMMNCs (190 million cells divided into 4 doses and resuspended into injection volumes as indicated) were resuspended and injected into the pinnae of recipient minipigs, n = 2. Two intradermal (0.1 ml per injection) and two subcutaneous (1 ml per injection) injections were administered to one minipig at t = 0, 7, 14 days. Two intradermal (0.1 ml per injection) and two subcutaneous (1 ml per injection) injections were administered to another minipig at t = 0, 21 days. Sera from minipigs were tested for circulating anti-donor antibodies at four weeks post-injection; these served as positive controls. Animals were humanely euthanized at endpoint.

#### Histopathology

To evaluate possible immune responses in the heart, hematoxylin-and-eosin staining was performed in sections obtained from the infarct (n = 3 slides per heart), the injection sites (n = 6 slides per heart), and remote LV myocardium (n = 3 slides per heart). Sections were analyzed by two independent pathologists blinded to treatment allocation. Analysis was graded according to the International Society for Heart & Lung Transplantation (ISHLT) guidelines, a system used in clinical practice to grade cardiac allograft rejection [39]. Additional histopathology features were assessed, including amount of calcification, granulation tissue, and necrosis. To measure myocyte cross-sectional area, sections obtained from the infarct border zone underwent immunostaining for troponin I (Abcam, Cambridge, MA) and wheat-germ agglutinin (Molecular Probes, Grand Island, NY) (to visualize cell borders); Alexa Fluor conjugated secondary antibodies (Molecular Probes) were used and counterstaining with DAPI (Molecular Probes) was performed. Sections were imaged using a confocal laser scan microscope (Leica Microsystems, Buffalo Grove, IL) and images were processed by Leica Application Suite software.

### Pivotal study

#### Cell culture

A freshly-explanted heart from one male farm pig was cultured as above, with the luciferase labeling step omitted, for generation of secondary CSps, which were harvested and suspended in CryoStor CS10 (BioLife Solutions, Bothell, WA) at a per animal dose of 150 million ICEs. CSps were frozen and stored at −80°C. Cells were thawed and filtered immediately prior to injection with a 160 µm nylon net filter to maintain a uniform maximum size.

#### Induction of MI and transendocardial cell injection

Twenty nine adult Yucatan minipigs (10±2 months; 14 male, 15 female) weighing 44±6 kg were studied. On day 0, minipigs underwent induction of MI by 100% occlusion of the mid-LAD for 3 hours, as described above. Eight weeks post-MI, minipigs received by random assignment: a) vehicle (CryoStor CS10); or b) CSps (150 million ICEs). An electroanatomical map of the LV was created and cells were injected as described above (Figure 2) by a single investigator blinded to treatment allocation, into 15 injection sites of 150 µl per injection. Eight weeks after injection, an electrophysiology study was performed (see Electrophysiology Study below); animals then underwent humane euthanasia, followed by explantation of the heart, brain, liver, kidneys, lungs, and spleen. In order to create a more clinically-realistic model of advanced chronic ischemic cardiomyopathy, a longer ischemia time (3 hours vs 2.5 hours), a later time-point of cell injection (8 weeks vs 3–4 weeks) and a longer follow-up (8 weeks vs 4 weeks) were used in the pivotal study compared to the dose optimization study.

#### Cardiac MRI

To assess efficacy, baseline (8 weeks post-MI, before transendocardial injections) and endpoint (8 weeks post-injection) contrast-enhanced cardiac MRI was performed, as described above [32]. Animals with LVEF>50% at baseline were excluded from the protocol and replaced (n = 3). MRIs were analyzed by an investigator blinded to treatment allocation. Scar mass, scar size, viable myocardium, total LV mass, LVEF, EDV, ESV, and indices of regional function (end-systolic wall thickness, wall thickening, wall motion) were measured.

#### Electrophysiology Study

To assess the inducibility of ventricular arrhythmias, an electrophysiology study with provocative testing was performed at t = 16 weeks. Programmed electrical stimulation of the right ventricle was performed as described [40]. In brief, eight ventricular drive beats (pacing cycle length 250 ms) were delivered. The first extra stimulus (S2) was set at 230 ms after the last pacing stimulus of the drive train (S1). S2 was progressively shortened in 10 ms decrements until the effective refractory period was reached. If no arrhythmia was induced, additional extra stimuli were introduced 170–200 ms after the previous extra stimulus. Inducibility of ventricular arrhythmias was recorded in a binary fashion.

#### Bloodwork

Complete blood count, chemistry panel, creatine kinase-MB (CKMB), cardiac troponin I (cTnI), C-reactive protein (CRP), and erythrocyte sedimentation rate (ESR) were analyzed at time points t = 0, 8 weeks (pre- and post-injection), 10 weeks, 16 weeks.

#### Atrial Natriuretic Peptide measurements

Venous blood was collected 8 and 16 weeks post-MI. Plasma levels of porcine α-ANP, which is similar to the human peptide [41], [42], were analyzed by two investigators blinded to treatment allocation. Venous blood was transferred to test tubes containing potassium EDTA (1.8 mg/mL). After gently mixing for 1 min, blood was centrifuged at 4°C and plasma was frozen and stored at −80°C until assay. Stored plasma was thawed, diluted with an equal volume of saline containing 40 mM HCl on ice, and centrifuged at 13,000× g at 4°C for 15 min. The resulting supernatant was loaded onto a Sep-Pak Plus C18 cartridge (Waters, Milford, MA, USA) that was prewashed sequentially with 5 mL each of chloroform and methanol, 15 mL each of 50% acetonitrile containing 0.1% trifluoroacetic acid (TFA), 0.1% TFA, and saline. After washing with 10 mL each of saline, 0.1% TFA, and 20% acetonitrile containing 0.1% TFA, the absorbed materials were eluted with 5 mL of 50% acetonitrile containing 0.1% TFA and lyophilized, as previously described [43]. The plasma extract was dissolved in 500 µL of radioimmunoassay (RIA) standard buffer, and a 100 µL aliquot of each sample was used for RIA. ANP is highly conserved among mammalian species [41]. The RIA used detects both porcine α-ANP and human α-ANP [44], and was performed using antiserum #125-8 at a final dilution of 400,000 [44]. A 50% inhibition value of tracer binding (IC_50_) was 10 fmol/tube, and α-ANP level was quantitatively measurable in a range of 1.0–100 fmol/tube.

#### Histocompatibility

Low-resolution swine leukocyte antigen typing was performed on the donor farm pig and all recipient minipigs as described above in the dose optimization Study.

#### Circulating anti-donor alloreactive antibodies

To evaluate humoral immune response, recipient pig serum was obtained at t = 8 weeks (prior to injection), 10 weeks and 16 weeks post-MI. Serum was screened with flow cytometry for circulating anti-donor IgG antibodies at all three time points by a single investigator blinded to treatment allocation. The allosensitization protocol described above in the dose optimization study provided positive and negative controls for comparison.

#### Histopathology

To evaluate immune responses in the heart, hematoxylin-and-eosin staining was performed in sections obtained from the infarct (n = 3 slides per heart), border zone (n = 6 slides per heart), remote myocardium within the LV (n = 3 slides per heart), left atrium (n = 1 slide per heart), right atrium (n = 1 slide per heart), and right ventricle (n = 1 slide per heart), as described above in the dose optimization study. Myocyte cross-sectional area was also analyzed as described above.

### Statistical analysis

Data are expressed as mean ± SD. Data were compared using paired t-test, independent t-test, Fisher's exact test, or ANOVA using a Bonferroni correction when applicable (SPSS Statistics 17.0, Chicago, IL). *p*<0.05 was considered statistically significant.

## Results

### Adverse events and mortality

In the engraftment study, 22/33 animals (67%) survived the initial MI and were included for analysis. There was no further mortality within the protocol, nor were there any adverse events.

In the dose optimization study, 16/24 animals (67%) with a baseline LVEF≤50% survived the initial MI and were included for analysis. There was no further mortality, nor were there any adverse events during the initial injection procedure in either group. One pig in the placebo group went into ventricular fibrillation during the endpoint electromechanical mapping procedure and was rapidly defibrillated with no other adverse events.

In the pivotal study, 22/29 animals (76%) with a baseline LVEF≤50% survived the initial MI and were included for analysis. There was no further mortality in these pigs. During the injection procedure, one pig in the placebo group went into ventricular fibrillation; the animal was defibrillated and completed the study with no further adverse events. One pig in the placebo group went into ventricular fibrillation during the endpoint electromechanical mapping procedure and was defibrillated with no other adverse events.

In the allosensitization study, there were no adverse events (0 of 3).

### Compatibility of CSps with the injection catheter

At concentrations ≥133 million ICEs in the form of CSps per ml, the injection solution was excessively viscous, precluding passage through the needle (Supporting Image S2). At all concentrations less than 133 million ICEs in the form of CSps per ml, cell viability was maintained (∼86% remain viable) after passage through the 27 g catheter needle. An isolated cell equivalent (ICE) is defined as the absolute number of cells in an aliquot of CSps. CSps are quantified as ICEs because any given cardiosphere is an aggregate that consists of a variable number of cells. In order to accurately quantify actual cell numbers, CSps are thus separated into the individual cells that make them up, then counted. Assuming an injection volume of 150 µl, a dose of 20 million ICEs as CSps per injection is the maximum which can be reliably resuspended. Due to the considerable time and resource constraints necessary for culturing this dose (300 million ICEs in the form of CSps per animal, delivered in 15 injections), our maximum feasible dose was set to 10 million ICEs in the form of CSps per injection (i.e., 150 million ICEs in the form of CSps per animal). The Myostar injection catheter is equipped with a 27 g needle (internal diameter of 210 µm) [45]. Porcine CSps can grow up to 200 µm in diameter [26]; ours were filtered to a maximum size of 160 µm. CSp size (Supporting Image S3) and viability (Supporting Image S2) were not compromised by passing through the injection catheter in the dose range tested.

### Histocompatibility

SLA haplotypes revealed complete Class I (SLA-1, SLA-2, SLA-3) and Class II (DRB1, DQB1, DQA) mismatches between the donor pig and all recipients in the dose optimization (Supporting Image S4) and pivotal (Supporting Image S5) studies.

### Engraftment Study

**Figure 3:**
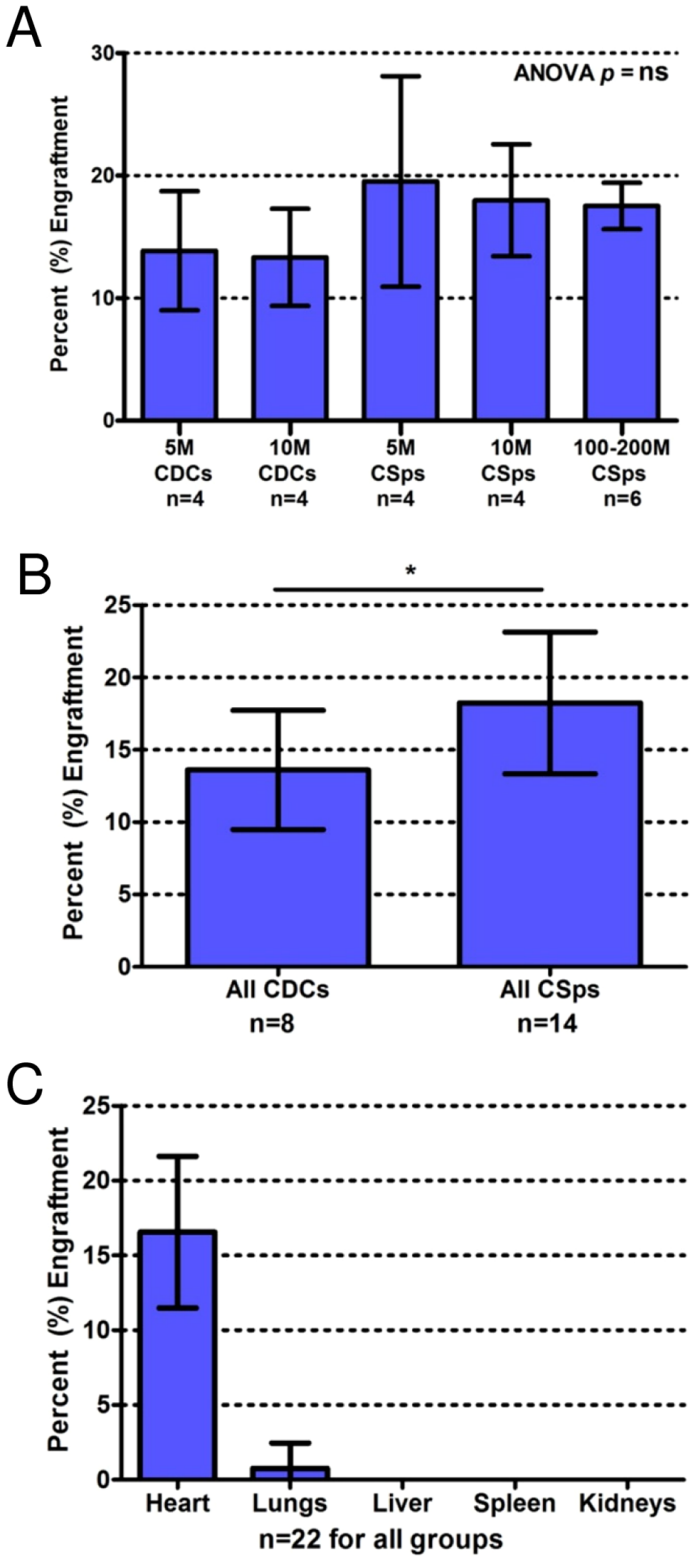
Cardiac percentage engraftment 24h post-injection. A) Comparison of cardiac engraftment between all five doses of CDCs and CSps. Increasing doses of either cell type do not increase percentage engraftment. However, since greater numbers of cells are being injected at higher doses, the absolute number of engrafted cells increases with higher cell doses. B) Pooled CSps had significantly greater 24 h engraftment than pooled CDCs (*p* = 0.036). C) There is little to no off-target engraftment (pooled CSps and CDCs, all doses); cardiac engraftment is significantly higher than pulmonary engraftment (*p*<0.0001).

Transendocardial injection of heart-derived cells resulted in robust cardiac retention, with minimal off-target engraftment (Figure 3). Twenty-four hour cardiac engraftment for 5 million CDCs was 13.9±4.9% (n = 4); 10 million CDCs was 13.3±4.0% (n = 4); 5 million ICEs delivered as CSps was 19.5±8.6% (n = 4); 10 million ICEs delivered as CSps was 18.0±4.6% (n = 4); and 100–200 million ICEs delivered as CSps was 17.5±1.9% (n = 6). There was no difference in percentage engraftment with increasing doses of CDCs (*p* = 0.87) or CSps (*p* = 0.84), nor was there any difference in percentage engraftment among the five groups (*p* = 0.34) (Figure 3A). However, 24 hour engraftment of pooled CSps (18.2±4.9%) was greater than that of CDCs (13.6±4.1%) (p = 0.036) (Figure 3B). Due to their superior percentage engraftment, we chose CSps for the dose-ranging study. There was minimal off-target engraftment of heart-derived cells (CSps and CDCs, 5–200×10^6^ cells) in the lungs (0.7±1.7%), liver (0%), spleen (0%), or kidneys (0%) (Figure 3C).

### Dose Optimization Study

#### Safety

**Figure 4:**
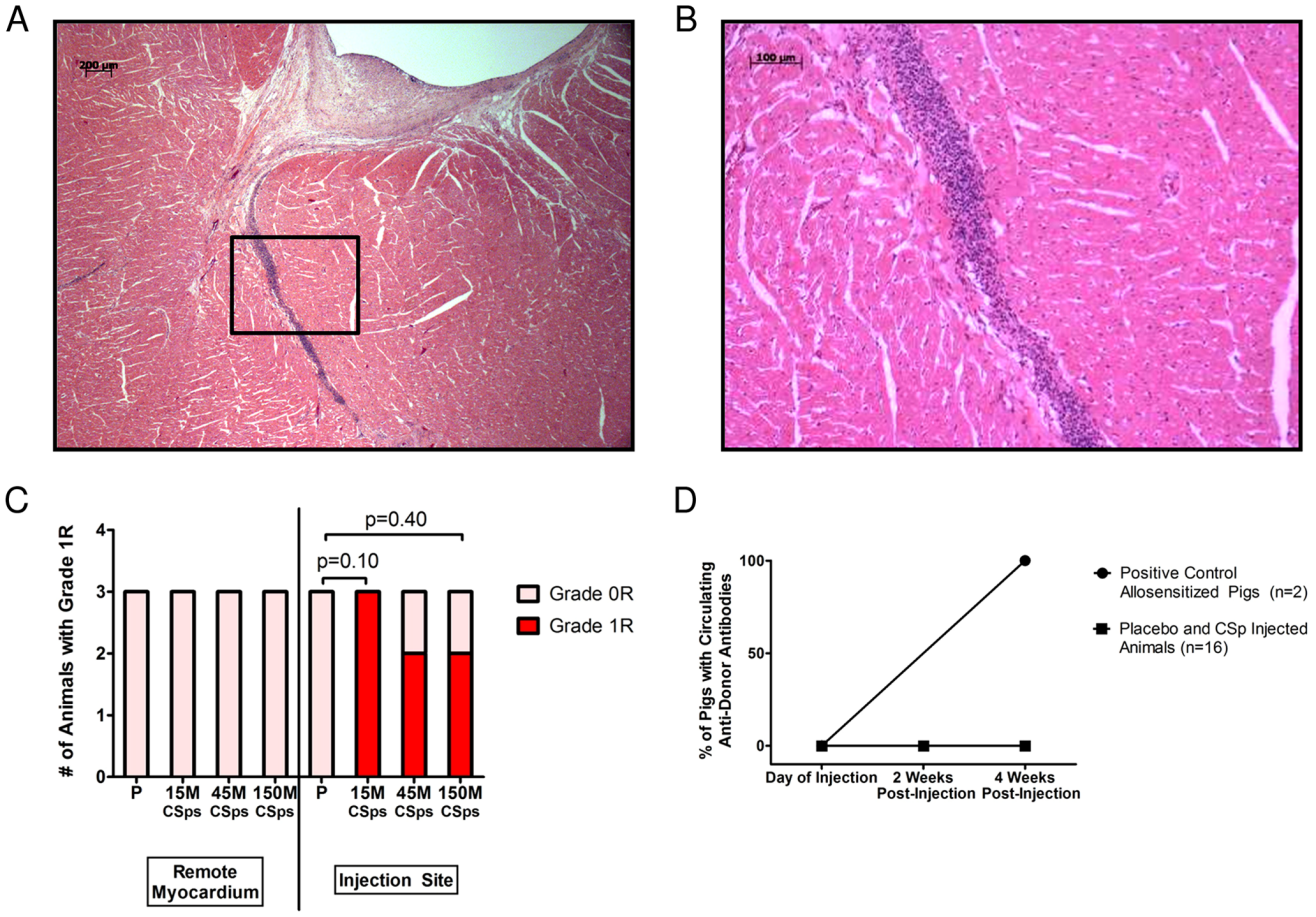
Immune response in the dose optimization study. A) After meticulous sampling of all heart sections, including the injection sites at the infarct border zone, this slide represents the worst case of immune reaction seen in all injected pigs in the dose optimization study. A lymphoplasmacytic cellular infiltration is observed near the injection site. B) High power image of boxed area in (A). C) Number of animals in dose optimization study showing Grade 0R (light pink) or 1R (red) histopathology in remote myocardium and injection sites. D) No circulating anti-donor antibodies could be detected in pigs that were transendocardially injected with allogeneic CSps at baseline and 4 weeks post-injection in the dose optimization study. High titers of circulating anti-donor antibodies were detected in minipigs 4 weeks post PBMMNC injection (subcutaneous and intradermal injections) that served as positive controls of allosensitization.

Blinded histopathological analysis revealed increased focal lymphoplasmacytic infiltration (Grade 1R) in the injection sites of minipigs injected with allogeneic CSps compared to those with placebo (Figure 4A–C). However, the incidence of Grade 1R histopathology was not dose-dependent. The infiltrating cells were localized within interstitial and perivascular spaces (Figure 4A,B); importantly, no foci of myocyte damage were detected. The remote myocardium was consistently clear of infiltrating inflammatory cells. Interestingly, there was a dose-dependent decrease in the amount of mineralization (calcium deposition) and granulomatous inflammation in CSp-injected animals compared to placebo. There were no other histologically-significant differences between CSp-injected and placebo-injected animals.

With regard to humoral memory response, no alloreactive antibodies were detected in any recipients of allogeneic CSps at any time point. In contrast, in the sensitized minipigs used as positive controls, high titers of circulating alloreactive IgG antibodies were detected four weeks post-injection (Figure 4D). Representative flow cytometry histograms are provided in Supporting Image S6.

#### Efficacy

**Figure 5:**
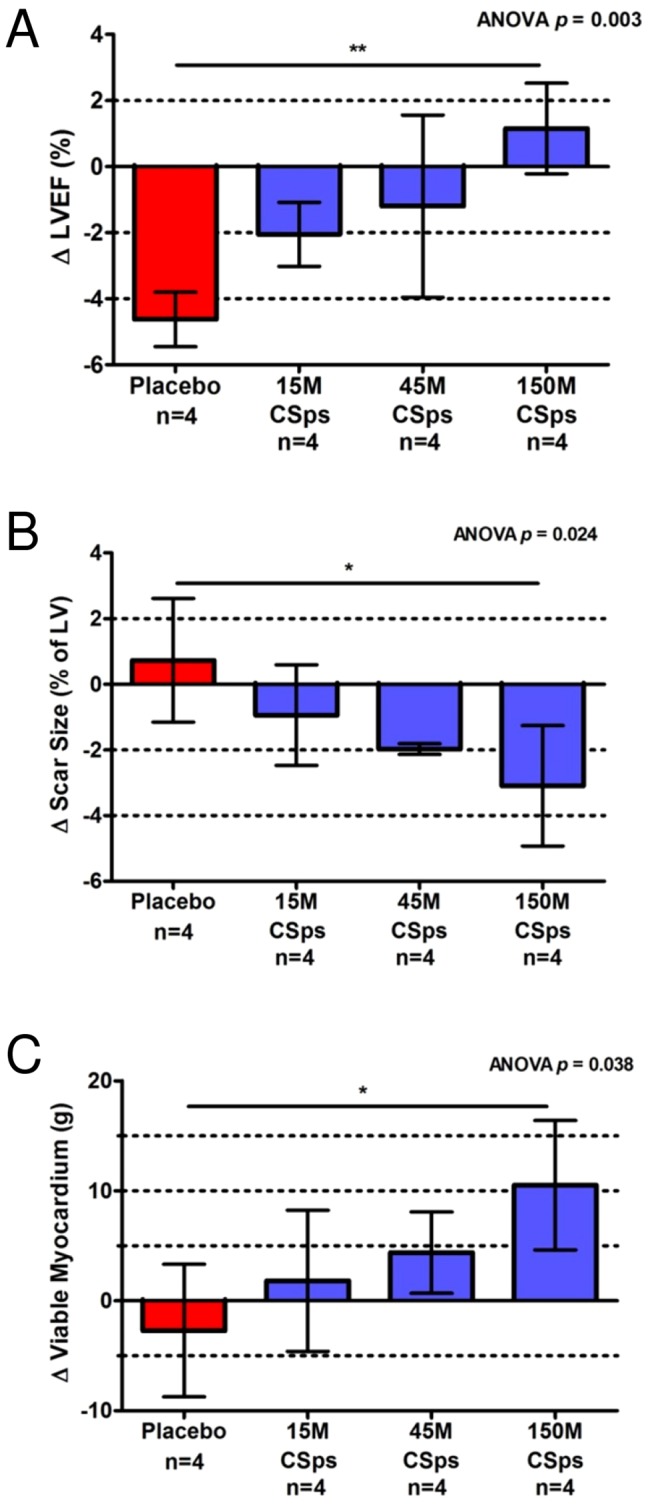
Dose-dependent functional and structural benefits after catheter-guided transendocardial injection of allogeneic CSps in dose optimization study. A) Comparison of LVEF treatment effect in all 4 dose optimization groups. B) Comparison of scar size treatment effect in all 4 dose optimization groups. C) Comparison of viable myocardial mass treatment effect in all 4 dose optimization groups.

To assess efficacy, minipigs underwent MRI before transplantation and four weeks later. Absolute values for MRI-measured parameters are displayed in Tables 1 & 2. Individual animal MRI indices are plotted in Supporting Image S7. All CSp doses attenuated remodeling; ESV increased in the control group (*p* = 0.015) but was preserved in all CSp groups (*p* = ns) (Table 1). The two higher CSp doses (45 & 150 million ICEs delivered as CSps) were effective in preserving LVEF (Figure 5A). No differences in regional function (systolic thickening, end-systolic thickness, and wall motion) of infarcted segments were observed between groups (Table 2).

**Table 1:** Summary of dose optimization MRI data by dose group.

MRI Index	Dose	Baseline	Endpoint	Paired t-test
		(t = 4 wk)	(t = 8 wk)	p value
Scar mass (g)	Placebo	8.3±0.8	8.5±1.3	ns
	15 mill CSps	8.8±1.9	8.6±2.9	ns
	45 mill CSps	7.9±2.9	7.0±3.1	0.023
	150mill CSps	10.0±2.2	8.4±1.3	ns
Scar size (% of LV)	Placebo	11.6±1.7	12.3±2.9	ns
	15 mill CSps	12.0±3.9	11.0±3.8	ns
	45 mill CSps	12.0±3.6	10.1±3.5	<0.001
	150mill CSps	13.4±1.8	10.3±1.8	0.043
Viable myocardium (g)	Placebo	63.6±5.3	60.9±8.0	ns
	15 mill CSps	70.4±12.2	72.2±6.3	ns
	45 mill CSps	58.0±5.3	62.4±5.9	ns
	150mill CSps	64.3±7.3	74.8±11.8	0.038
LV mass (g)	Placebo	71.9±4.7	69.4±7.2	ns
	15 mill CSps	79.2±11.4	80.8±5.9	ns
	45 mill CSps	65.9±7.8	69.3±8.8	ns
	150mill CSps	74.3±8.9	83.2±12.2	0.039
LVEF (%)	Placebo	47.6±2.4	42.9±2.4	0.002
	15 mill CSps	43.7±5.8	41.7±5.6	0.024
	45 mill CSps	45.2±3.1	44.0±4.8	ns
	150mill CSps	47.5±3.0	48.6±1.8	ns
EDV (ml)	Placebo	71.1±10.2	79.6±10.5	ns
	15 mill CSps	81.2±11.0	88.6±13.7	ns
	45 mill CSps	65.8±14.4	74.1±12.6	ns
	150mill CSps	73.2±5.8	79.6±11.4	ns
ESV (ml)	Placebo	37.4±6.0	45.6±7.7	0.015
	15 mill CSps	45.7±7.5	51.3±6.8	ns
	45 mill CSps	36.2±8.9	41.7±9.0	ns
	150mill CSps	38.4±1.8	40.9±5.4	ns
Body Weight Gain (kg)	Placebo	n/a	6.6±1.9	ns (ANOVA & Ind t-test)
	15 mill CSps	n/a	8.1±3.0	
	45 mill CSps	n/a	6.5±2.9	
	150mill CSps	n/a	8.1±2.2	
HR during MRI (bpm)	Placebo	n/a	119±10	ns (ANOVA & Ind t-test)
	15 mill CSps	n/a	109±17	
	45 mill CSps	n/a	111±15	
	150mill CSps	n/a	108±13	

**Table 2:** Summary of dose optimization MRI data by dose group.

MRI Index	Dose	Endpoint (t = 8 wk)	p value (ANOVA)
Wall thickness (mm)	Placebo	6.2±1.2	ns
	15 mill CSps	6.0±2.4	ns
	45 mill CSps	5.2±1.7	ns
	150mill CSps	6.0±1.2	ns
Wall thickening (%)	Placebo	14.7±33.8	ns
	15 mill CSps	22.3±22.3	ns
	45 mill CSps	7.8±25.9	ns
	150mill CSps	11.5±27.1	ns
Wall motion (mm)	Placebo	2.1±1.3	ns
	15 mill CSps	2.7±1.5	ns
	45 mill CSps	2.1±1.6	ns
	150mill CSps	2.3±1.5	ns

**Figure 6:**
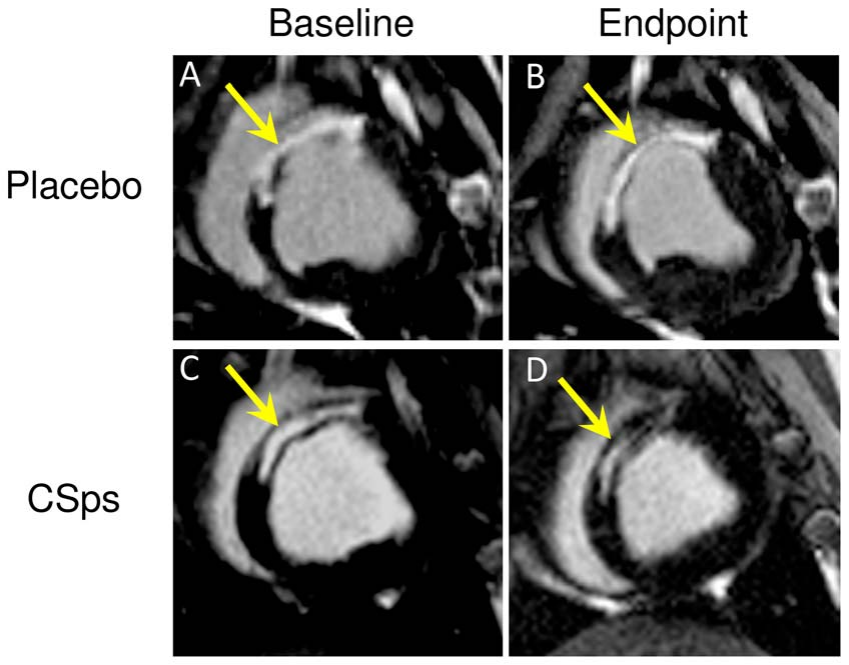
Myocardial regeneration after catheter-guided transendocardial injection of allogeneic CSps in dose optimization Study. Representative short-axis slices of contrast-enhanced cardiac MRIs at baseline (left) and 4 weeks later (right) in a control pig (top row) and a CSp-treated pig (45 million ICEs delivered as CSps; lower row). Infarct scar tissue is evident by areas of hyperintensity (white) whereas viable myocardium appears dark. Arrows point to infarct scar.

In terms of regenerative efficacy, the key parameters are viable myocardial mass and scar size [6]. Figure 6 shows representative images from a control animal (A,B) and a CSp-treated animal (C,D) at baseline (A,C) and 4 weeks later (B,D). The scar is hyperenhanced (white), while viable myocardium appears black. In the control animal (A,B), the scar does not become smaller over time, nor does the amount of viable myocardium appear to increase. In contrast, the CSp-treated heart (C,D) visibly undergoes scar shrinkage as well as an increase in viable mass over the month after treatment. Data such as these, when analyzed volumetrically and pooled, yield the average results plotted in Figures 5B (change in scar size) and 5C (change in viable myocardial mass). While increasing CSp doses led to progressively greater improvement, the 150 million CSp dose was superior in increasing viable myocardium (Figure 5C) and in reducing scar size (Figure 5B), and was thus chosen for further testing in our pivotal study (see below).

**Figure 7:**
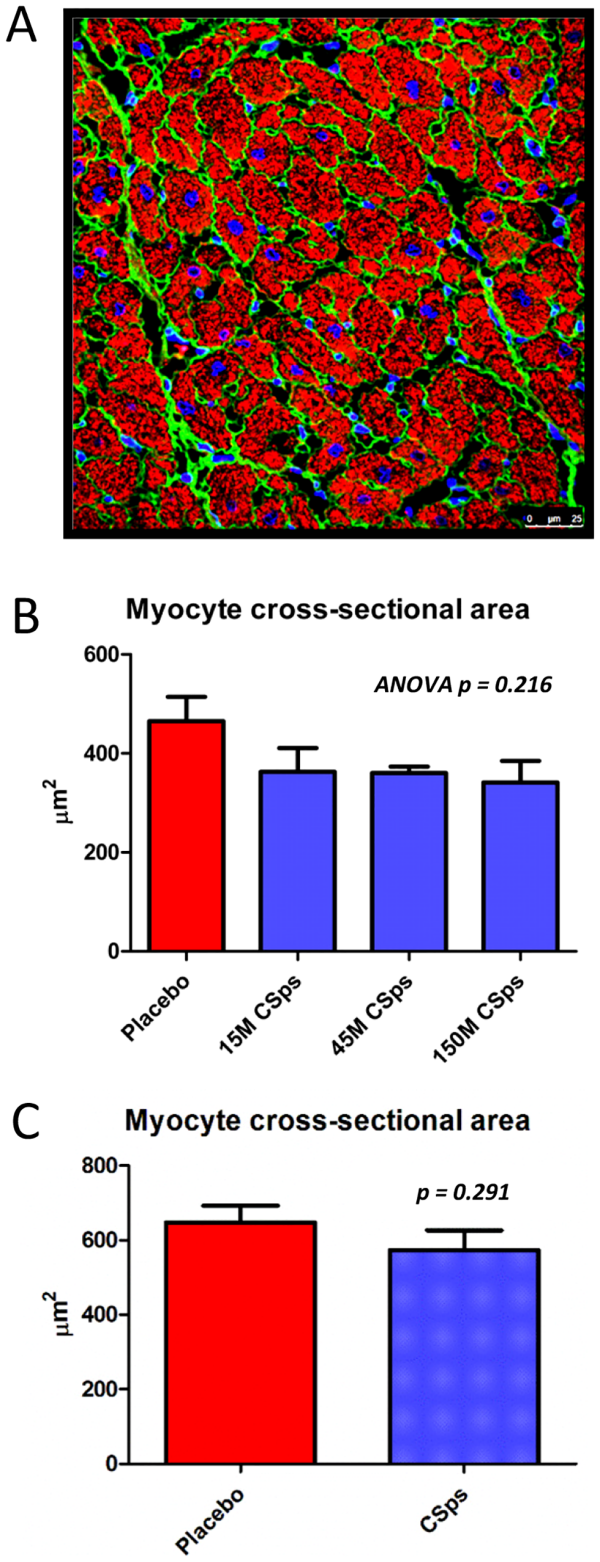
Myocyte cross-sectional area. A) Representative section showing cardiomyocyte cross-sectional area at infarct border zone (injection site). B) Dose optimization study cross-sectional area was measured and compared, showing similar myocyte sizes across all 4 groups (p = ns). C) Pivotal study cross-sectional area was measured and compared, showing similar myocyte sizes in placebo and cell-treated groups (p = ns).

An increase in viable myocardium by MRI may reflect either more myocytes (regeneration) or larger myocytes (hypertrophy) [32]. Cardiomyocyte cross-sectional area was similar in CSp-treated minipigs and controls (Figure 7B), thus excluding hypertrophy as a contributor to the increase in viable myocardium observed after cell therapy.

### Pivotal Study

#### Safety

**Figure 8:**
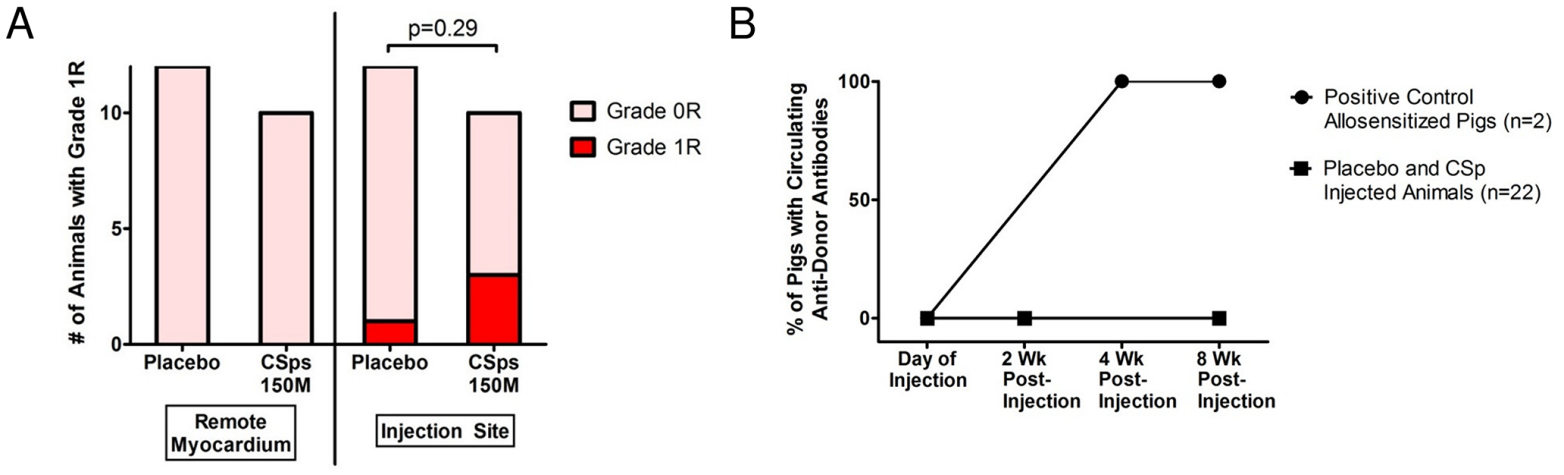
Immune response in the pivotal study. A) Number of animals in pivotal study showing Grade 0R (light pink) or 1R (red) histopathology in remote myocardium and injection sites. B) No circulating anti-donor antibodies could be detected in pigs that were transendocardially injected with allogeneic CSps 2 or 8 weeks post-injection in the pivotal study. High titers of circulating anti-donor antibodies were detected in minipigs 4 weeks post PBMMNC injection (subcutaneous and intradermal injections) that served as positive controls of allosensitization.

Blinded histopathological analysis revealed no abnormalities in the brain, kidneys, liver, lung, or spleen related to heart-derived cell injection. In contrast to the dose optimization study, cardiac histopathology did not reveal increased lymphoplasmacytic infiltration in the border zone of minipigs injected with allogeneic CSps 8 weeks post-injection (Figure 8A). This difference may be attributed to a transient mononuclear infiltration that, while detectable one month post-injection (endpoint of the dose optimization study), had fully resolved by 8 weeks post-injection (endpoint of the pivotal study). No foci of inflammation-associated myocyte damage were detected. There was a decreased occurrence of mineralization/calcium deposition and granulomatous inflammation in cell-treated animals, similar to what was observed in the dose optimization study.

With regard to a humoral memory response, no alloreactive antibodies were detected in any recipients of allogeneic CSps at any time point (Figure 8B).

Comparisons of 39 blood parameters showed no significant differences in any inflammatory or immunological markers (total white blood cell count, CRP, ESR) or injury-related markers (cTnI, CKMB) at all time-points. Endpoint neutrophil count was significantly higher in cell-treated animals; however, this was due to one outlier pig who suffered a forelimb lameness during the study. All other animals in the cell-treated group had a neutrophil count within normal limits.

As in our previous porcine studies [26], [30], all animals in both groups exhibited inducible arrhythmias at endpoint, with no difference between placebo and cell-treated groups.

#### Efficacy

**Figure 9:**
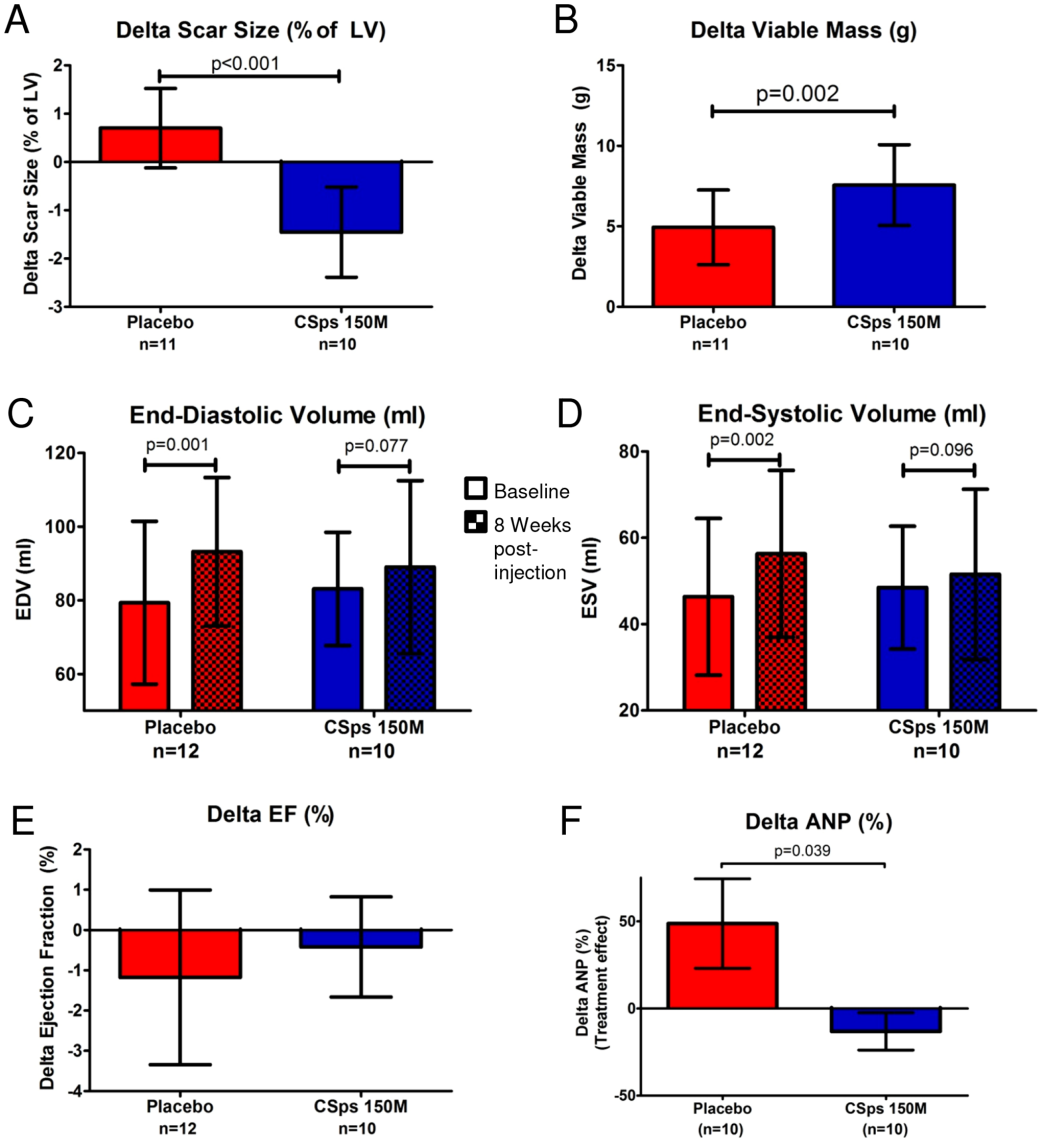
Dose-optimized transendocardial injection of allogeneic CSps in the pivotal study attenuated remodeling, decreased scar size and increased viable myocardium in a porcine model of chronic ischemic cardiomyopathy. A) Comparison of scar size treatment effect in both placebo and cell-treated pivotal study groups. B) Comparison of viable myocardium treatment effect in both placebo and cell-treated pivotal study groups. C) End-diastolic volume measurements at baseline and 8 weeks post-injection in both placebo and cell-treated pivotal study groups. D) End-systolic volume measurements at baseline and 8 weeks post-injection in both placebo and cell-treated pivotal study groups. E) Comparison of LVEF treatment effect in both placebo and cell-treated pivotal study groups. F) Comparison of plasma ANP treatment effect in both placebo and cell-treated pivotal study groups.

To assess efficacy, minipigs underwent MRI before CSp transplantation and eight weeks later. MRIs were analyzed in a blinded manner; results are displayed in Tables 3 & 4. Individual animal MRI indices are plotted in Supporting Image S7. No significant improvement in global (LVEF) (Figure 9E) or regional (systolic thickening, end-systolic thickness, wall motion) LV function could be observed in cell-treated animals compared to controls (Table 4). The fact that EF in control pigs in the pivotal study remained relatively stable over the follow-up period (between weeks 8–16 post-MI), while it decreased in control pigs in the dose-response study (between weeks 3–7 post-MI) is consistent with the natural history of porcine ischemic cardiomyopathy; EF drops in the first weeks post-MI and remains relatively stable thereafter [28], [29], [46]. Despite the lack of an effect of cell therapy on EF in this protocol, minipigs treated with allogeneic CSps displayed attenuation of structural adverse remodeling. EDV and ESV increased significantly in the placebo group but not in the CSp group over the 8 week follow-up (Figure 9C,D). In addition, while plasma ANP increased in the placebo arm between baseline and endpoint, ANP levels remained stable in cell-treated pigs. This finding supports the global hemodynamic significance of the observed attenuation of LV remodeling (Figure 9F).

**Table 3:** Summary of pivotal study MRI data by dose group.

MRI Index	Treatment	Baseline (t = 8 wk)	Endpoint (t = 16 wk)	Paired t-test p value
Scar mass (g)	Placebo	6.1±1.9	7.2±2.4	<0.001
	CSps	6.7±3.1	6.4±2.6	ns
Scar size (% of LV)	Placebo	10.9±2.3	11.5±2.4	0.026
	CSps	11.1±3.2	9.5±2.7	<0.001
Viable myocardium (g)	Placebo	50.0±6.3	54.7±6.5	<0.001
	CSps	51.9±6.8	60.0±6.9	<0.001
LV mass (g)	Placebo	56.1±7.6	61.9±8.1	<0.001
	CSps	58.6±9.2	66.4±8.9	<0.001
LVEF (%)	Placebo	42.1±7.0	40.9±7.7	ns
	CSps	43.6±5.2	43.2±5.1	ns
EDV (ml)	Placebo	80.8±21.7	93.2±20.1	0.001
	CSps	81.7±15.5	89.0±23.5	ns
ESV (ml)	Placebo	48.0±18.3	56.3±19.3	0.002
	CSps	46.7±13.6	51.5±19.7	Ns
Body weight gain (kg)	Placebo	n/a	+12.5±3.8	ns (Ind t-test)
	CSps	n/a	+14.0±4.7	
HR during MRI (bpm)	Placebo	n/a	112±13	ns (Ind-t-test)
	CSps	n/a	99±18	

**Table 4:** Summary of pivotal study MRI data by dose group.

MRI Index	Treatment	Endpoint (t = 16 wk)	p value (Ind t-test)
Wall thickness (mm)	Placebo	4.2±1.6	ns
	CSps	4.6±2.3	ns
Wall thickening (%)	Placebo	12.6±23.1	ns
	CSps	10.9±26.0	ns
Wall motion (mm)	Placebo	2.2±1.4	ns
	CSps	2.3±1.7	ns

In terms of regenerative efficacy, CSp-treated animals benefited from a significant increase in viable myocardium (Figure 9B) and concomitant decrease in scar size compared to placebo (Figure 9A). Importantly, cardiomyocyte cross-sectional area was similar in CSp-treated minipigs and controls (Figure 7C), excluding hypertrophy as a contributor to the increase in viable myocardium observed after cell therapy.

## Discussion

We have tested, systematically, the feasibility, safety and efficacy of percutaneous intramyocardial delivery of heart-derived cells. We first optimized dosing and delivery protocols for catheter-guided transendocardial injections. We then tested the safety and efficacy of allogeneic CSps in a clinically-relevant large animal model of chronic ischemic cardiomyopathy. The salient findings are:

1. Catheter-guided delivery of heart-derived cell products produces robust cardiac engraftment, with minimal off-target engraftment. While escalating doses of CSps and CDCs do not affect percentage engraftment, injection of CSps results in more robust percentage engraftment compared to CDCs.
2. Delivery of allogeneic CSps is safe, inducing a transient, mild local immune response with no signs of systemic immunogenicity or toxicity.
3. Dose-optimized allogeneic CSp injection produces functional benefits and therapeutic regeneration in a porcine model of chronic ischemic cardiomyopathy.

While most cell therapy studies to date have focused on intracoronary delivery in acute or convalescent MI patients [22], chronic ischemic cardiomyopathy patients represent a growing target population. The chronic setting is likely more difficult to treat with cell therapy because adverse cardiac remodeling has already progressed, and local homing signals are reduced [16]. These attenuating factors might, in principle, be counteracted by more effective and targeted delivery of cells. To that end, intramyocardial delivery has been shown to result in higher cardiac retention compared to intracoronary or systemic approaches, while implementation of electromechanical mapping enables targeted delivery of cells to viable myocardium in the border zone of chronic scars. In addition, in the vast majority of preclinical and clinical studies of transendocardial delivery of cell types grown from other organs, dosing has not been systematic; instead, it has been guided by feasibility and accessibility rather than purposeful optimization.

Here, we demonstrate that percutaneous transendocardial delivery of heart-derived cell products in the infarct border zone results in robust cardiac engraftment of up to 20%, higher than with any other previously-reported delivery method [3]. CSps outperform other stem cell types, where 24 hour engraftment is generally <10%, regardless of delivery route [13]. Injection of CSps results in more robust short-term engraftment compared to CDCs. The latter may be attributable to the larger size of CSps (allowing for greater passive entrapment of CSps within the myocardium), as well as to upregulation of adhesion molecules on the CSp surface [25].

CSps are self-organized spherical clusters of heart-derived cells, characterized by increased stemness [25]. Due to their larger size of 40–150 µm, CSps may not be safe to administer intracoronarily, as they would likely embolize in the microvasculature. In previous reports, we showed that IM delivery of CSps disproportionately boosts cardiac function in small and large animal models of ischemic cardiomyopathy compared with monolayer-cultured CDCs [25], [26].

We have also shown that injection of allogeneic CDCs and CSps in a stringent rat model of allotransplantation post-MI is safe, inducing a transient local immune response [47], [48]. Allogeneic cell therapy is desirable because it circumvents the logistic, economic, and timing constraints associated with autologous cell therapy. It also enables a highly-standardized off-the-shelf product to be generated from a universal donor pool. Here we show that transendocardial injection of allogeneic CSps in a large-animal model of chronic ischemic cardiomyopathy induces a mild local immune response with no signs of systemic immunogenicity or toxicity. No circulating anti-donor antibodies were detected in any of the treated pigs, giving reason for optimism with regard to the immunogenicity of allogeneic CSps in humans. The latter is important since patients with advanced ischemic cardiomyopathy may also be candidates for solid organ transplantation, such that highly-immunogenic agents are best avoided.

By design, the dose optimization study preceded the pivotal study. Because the two studies were not performed contemporaneously, direct comparison of the outcome is not straightforward. Nevertheless, the beneficial effects of cardiospheres appear to be blunted in the pivotal study in comparison to the dose optimization study. The benefits may genuinely be more pronounced with treatment 4 weeks post-MI than 8 weeks post-MI: intervention in the early phases of remodeling and infarct healing may be more effective compared to intervention after healing is complete, but this possibility remains conjectural. EF drops in the first weeks post-MI and remains relatively stable thereafter [28], [29], [46]. There is a minimal, albeit detectable, decline in EF that occurs between 4 and 12 weeks post-MI, where increased myocardial fibrosis is present [49]. This may blunt the effects of cell therapy, though intervention up to 8 weeks post-MI may still be beneficial in myocardial regeneration, as evidenced by the present study. Xiong et al [50] recently studied human induced pluripotent stem cells in an acute infarct model with a 4 week followup period. Even though the Xiong model is different, our outcomes appear to be at least as effective, and sometimes even better, in improving infarct size. Interestingly, the LVEF in Xiong et al increased by approximately +6% in both the placebo and cell-treated arms between 1 and 4 weeks post-MI, while in our study, the LVEF *fell* in the placebo arm of both the dose optimization and pivotal studies (approximately −5% and −2%, respectively), while increasing or staying the same in the 150M dose treated arms (approximately +1% and +0%, respectively). Moreover, the animal model used by Xiong et al used juvenile Yorkshire swine whose cardiac physiology may differ from those of older minipigs [51], [52]. Lastly, the animals in the paper by Xiong et al were immunosuppressed, unlike those in our study.

Finally, we show that dose-optimized percutaneous transendocardial injection of allogeneic CSps, tested in a randomized, blinded, placebo-controlled manner, attenuates remodeling, decreases scar size and increases viable myocardium. CSps are rich in matrix metalloproteinases [26], which could contribute to dissolution of scar tissue. The increase in viable myocardium did not result from myocyte hypertrophy, and likely occurs through stimulation of endogenous protective (anti-apoptotic), and regenerative mechanisms (upregulation of cardiomyocyte cycling, increased recruitment of endogenous stem cells [47], [53], [54], [55], [56]). The decreases in scar size, mirrored by increases in viable myocardium, resemble the changes seen in human subjects treated with CDCs [6] and are indicative of therapeutic regeneration. It should be noted that we observed greater benefits, especially with regard to EF, in the dose optimization study when CSps were injected 3–4 weeks post-MI, compared to the pivotal study where CSps were injected 8 weeks post-MI. Whether this means that cell therapy is more effective in the setting of convalescent MI compared to chronic ischemic cardiomyopathy remains to be further investigated.

### Limitations

Our study has several limitations. First, we only quantified 24 hour cardiac retention but not long-term engraftment. However, long-term engraftment is vanishingly low regardless of delivery method [4]; early cardiac cell retention appears to be a better predictor of long-term functional benefit [11], [12], [13]. Second, while we measured total 24 hour cardiac engraftment, we did not investigate the distribution of the transplanted cells within the host myocardium. While luciferase might conceivably be measured from nonviable cells, this is unlikely given the short 3 hour half-life of luciferase and the requirement for ATP [57]. Third, our study does not provide any mechanistic insight into the regenerative effects of CSps. Fourth, we have not thoroughly validated the use of MRI to assess therapeutic regeneration after CSp IM injection, as we have done with CDC intracoronary delivery [32]. However, the similarity between the two cell products with regard to mechanism of action [25] gives some reassurance that MRIs will be similarly interpretable in the current protocols. Fifth, as with all animal studies, there is the potential that the increase in LV mass in each of the chronic MI groups could be confounded by growth of the animal; however, if this were the case then all parameters should be similarly and linearly changed. Finally, the normal range of porcine-ANP has not been established, nor did we measure porcine-ANP in normal minipigs. Therefore, we are only able to assay relative changes within the two groups (CSp vs placebo) from baseline to endpoint.

## Conclusions

Transendocardial injection of heart-derived cell products results in robust short-term cardiac engraftment (better with CSps than CDCs). Delivery of allogeneic CSps is safe, inducing a transient, mild local immune response with no signs of systemic immunogenicity or toxicity. Dose-optimized injection of CSps attenuates cardiac dilatation, decreases scar size, and increases viable myocardium. The increases in viable myocardium, mirrored by decreases in scar, are consistent with genuine regeneration.

## Supporting Information

Supporting Image S1 Ex vivo luciferase assay. A) Timeline of steps in performing ex vivo luciferase assay. B) Representative standard curve created by measuring luciferase signal from known numbers of transduced cells.
(TIF)

Supporting Image S2 Determination of maximal feasible dose. Cells were dosed on a per pig basis. Total injection volume was predetermined at 1.5 ml (150 µl per injection ×10 injections). Viability and cell number were compared before and after injection through the Myostar injection catheter. At doses higher than 133 million ICEs delivered as CSps/ml, the solution could not be extruded through the injection catheter.
(TIF)

Supporting Image S3 Size distribution of CSps. Porcine cells were qualitatively analyzed for size distribution, both pre- and post-injection through the Myostar catheter. Size distribution of cells remained similar pre- and post-injection, indicating that CSp clusters remained intact during passage through the catheter.
(TIF)

Supporting Image S4 Summary of SLA haplotype mismatches between stem cell donor farm pig and each recipient minipig in dose optimization study. Donor pig inferred low-resolution SLA haplotype is Lr-26.23/26.23 [35], [36].
(TIF)

Supporting Image S5 Summary of SLA haplotype mismatches between stem cell donor farm pig and each recipient minipig in pivotal study. Donor pig inferred low-resolution SLA haplotype is Lr-35.12/35.23.
(TIF)

Supporting Image S6 Assessment of humoral memory response in pivotal study. Levels of circulating IgG anti-donor antibodies were measured in pig serum samples with flow cytometry. A) High titers of circulating anti-donor antibodies were detected in minipigs 4 weeks post PBMMNC injection (subcutaneous and intradermal injections) that served as positive controls with allosensitization. B) Representative sample of cell-injected pig. No circulating anti-donor antibodies could be detected in any pigs that were transendocardially injected with allogeneic CSps or placebo.
(TIF)

Supporting Image S7 Individual animal MRI data from both dose optimization and pivotal studies, plotted together. Error bars show 95% confidence interval. Time points are given as number of weeks post-MI. A) Scar size decreases with cell injection and increases with placebo in both the dose optimization and pivotal studies. B) Viable left ventricular mass increases with cell injection and decreases with placebo in the dose optimization study. Viable left ventricular mass increases with both cell injection and placebo in the pivotal study, though the increase is less dramatic with placebo. C) End diastolic volume increases with both cell injection and placebo. In the pivotal study, endpoint EDV increases significantly with placebo only, while EDV increases non-significantly with cell injection, corresponding with preservation of left ventricular remodeling. D) End systolic volume increases with both cell injection and placebo. In both the dose optimization and pivotal studies, endpoint ESV increases significantly in placebo while cell injection maintains ESV, corresponding with preservation of left ventricular remodeling. E) Left ventricular ejection fraction decreases with placebo in both the dose optimization and pivotal studies; the decrease is significant in the dose optimization study. Ejection fraction increases non-significantly with cell injection in the dose optimization study and decreases non-significantly with cell injection in the pivotal study.
(TIF)
