# Supplementary figures and images for: Allogeneic Cardiospheres Delivered via Percutaneous Transendocardial Injection Increase Viable Myocardium, Decrease Scar Size, and Attenuate Cardiac Dilatation in Porcine Ischemic Cardiomyopathy
Source: PLoS One. 2014 Dec 2;9(12):e113805. doi: 10.1371/journal.pone.0113805 (PMC4251970; doi:10.1371/journal.pone.0113805)

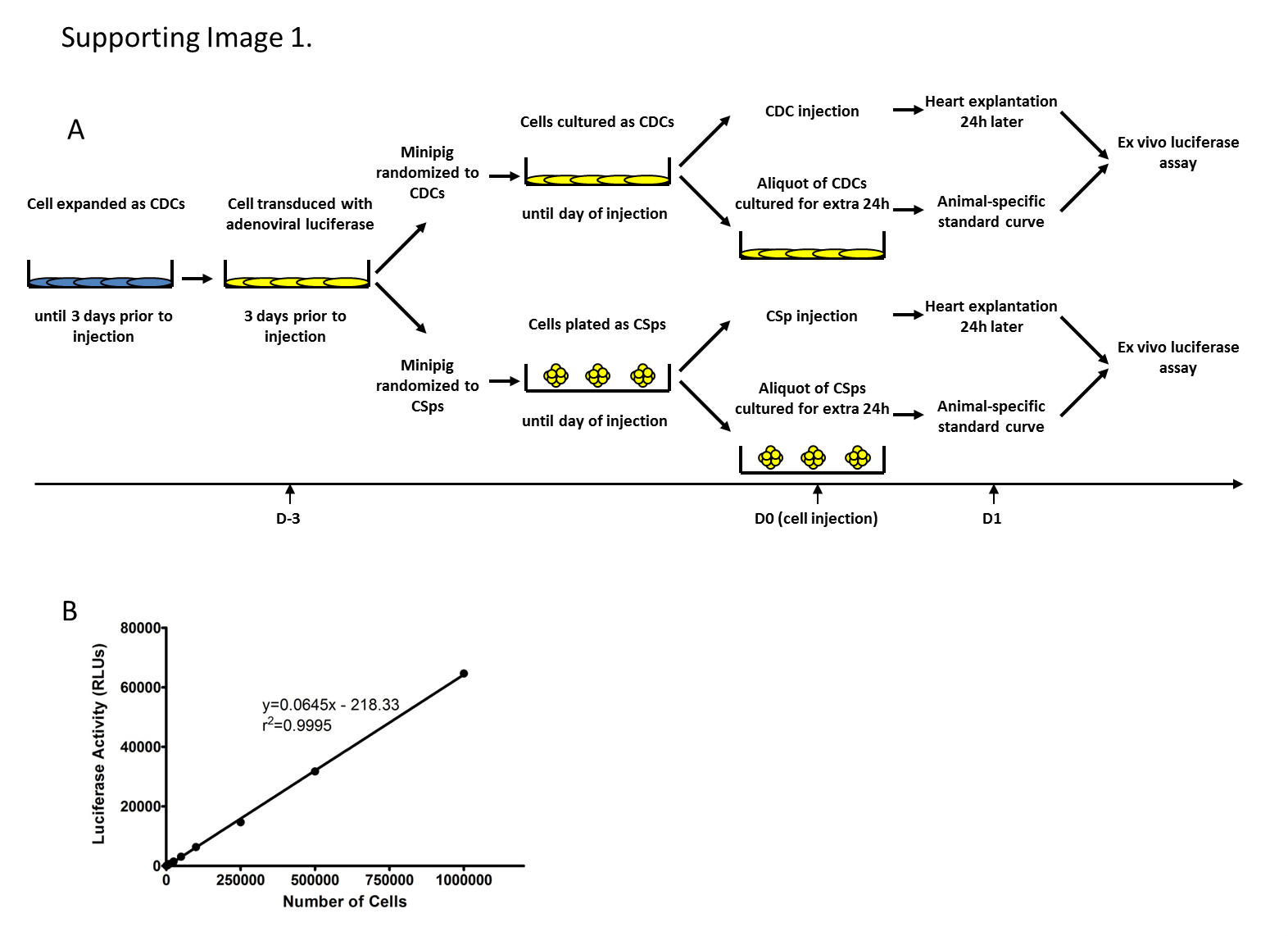

Supplement: Supporting Image S1 — Ex vivo luciferase assay. A) Timeline of steps in performing ex vivo luciferase assay. B) Representative standard curve created by measuring luciferase signal from known numbers of transduced cells. (TIF) [file pone.0113805.s001.tif]

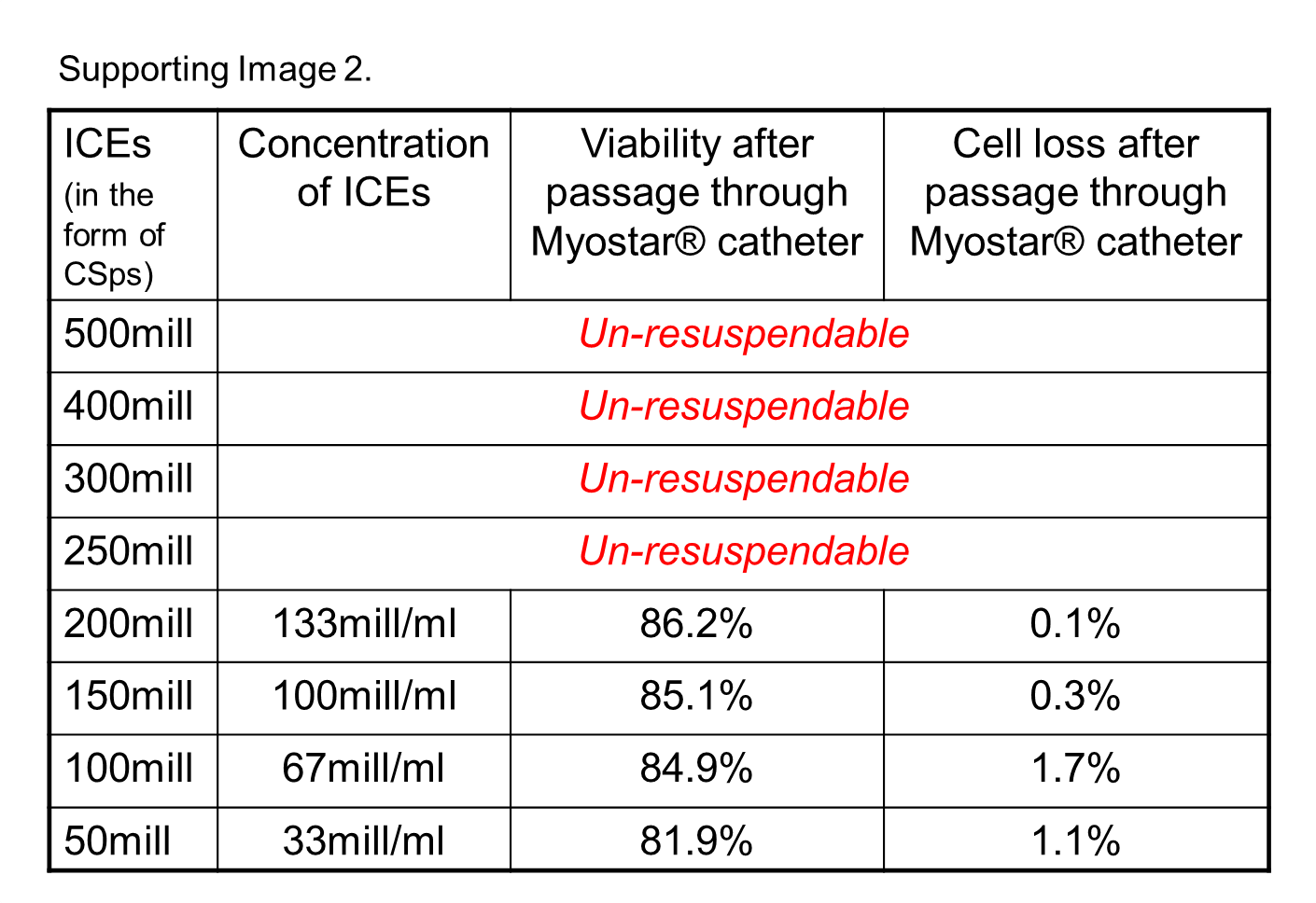

Supplement: Supporting Image S2 — Determination of maximal feasible dose. Cells were dosed on a per pig basis. Total injection volume was predetermined at 1.5 ml (150 µl per injection ×10 injections). Viability and cell number were compared before and after injection through the Myostar injection catheter. At doses higher than 133 million ICEs delivered as CSps/ml, the solution could not be extruded through the injection catheter. (TIF) [file pone.0113805.s002.tif]

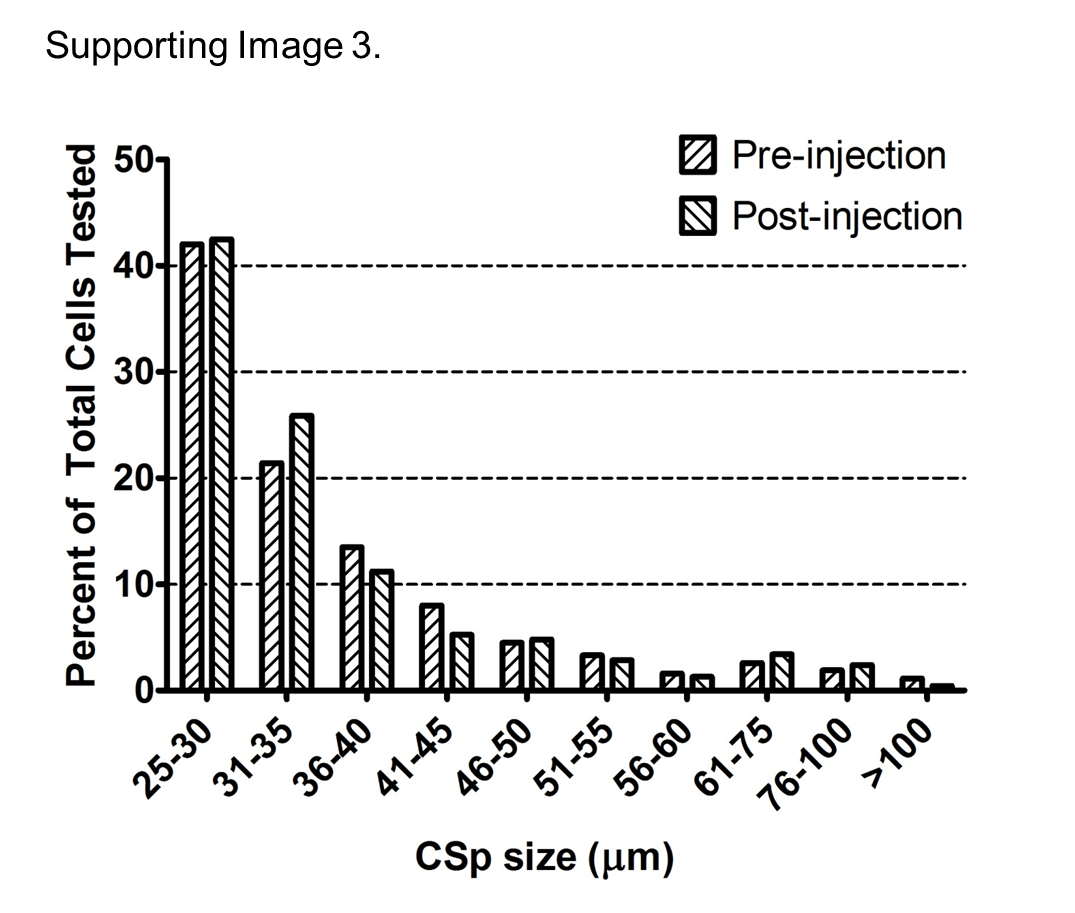

Supplement: Supporting Image S3 — Size distribution of CSps. Porcine cells were qualitatively analyzed for size distribution, both pre- and post-injection through the Myostar catheter. Size distribution of cells remained similar pre- and post-injection, indicating that CSp clusters remained intact during passage through the catheter. (TIF) [file pone.0113805.s003.tif]

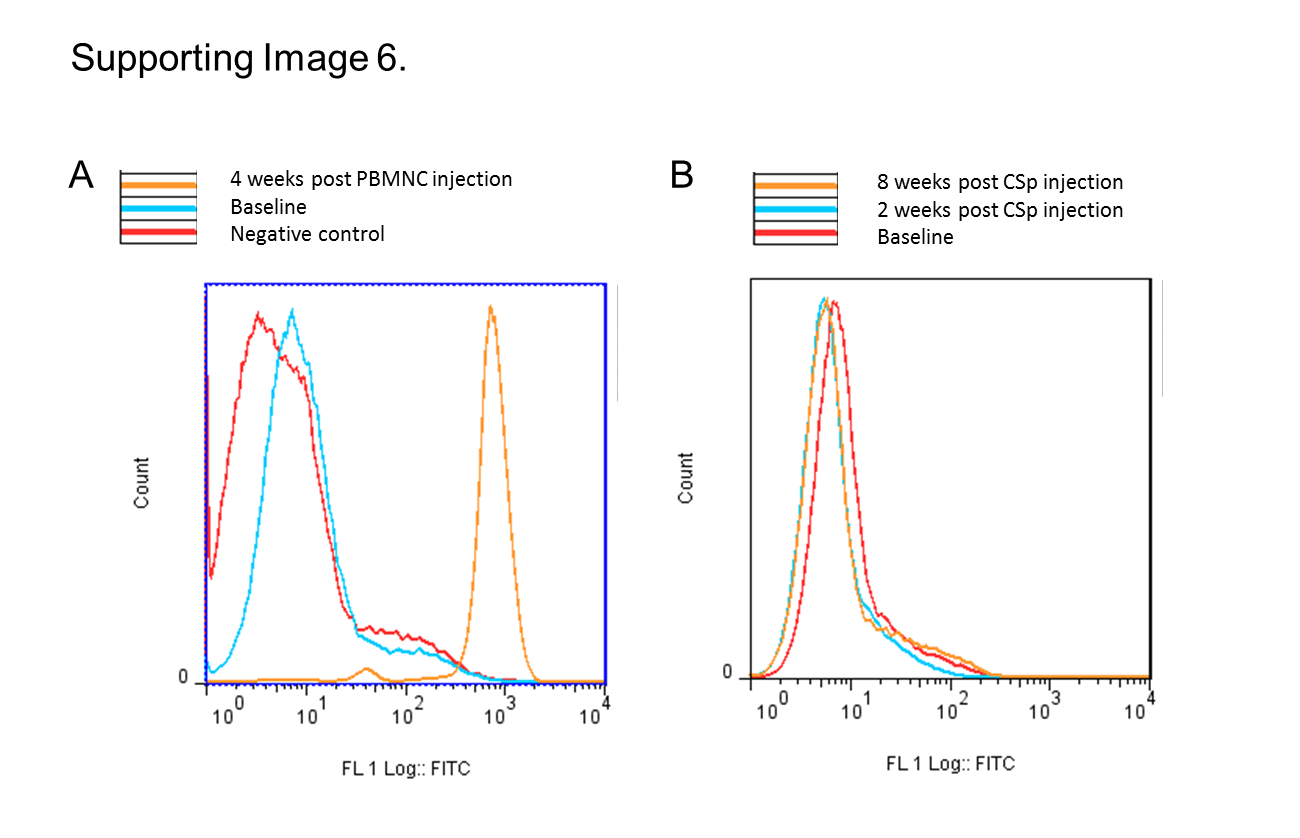

Supplement: Supporting Image S6 — Assessment of humoral memory response in pivotal study. Levels of circulating IgG anti-donor antibodies were measured in pig serum samples with flow cytometry. A) High titers of circulating anti-donor antibodies were detected in minipigs 4 weeks post PBMMNC injection (subcutaneous and intradermal injections) that served as positive controls with allosensitization. B) Representative sample of cell-injected pig. No circulating anti-donor antibodies could be detected in any pigs that were transendocardially injected with allogeneic CSps or placebo. (TIF) [file pone.0113805.s006.tif]

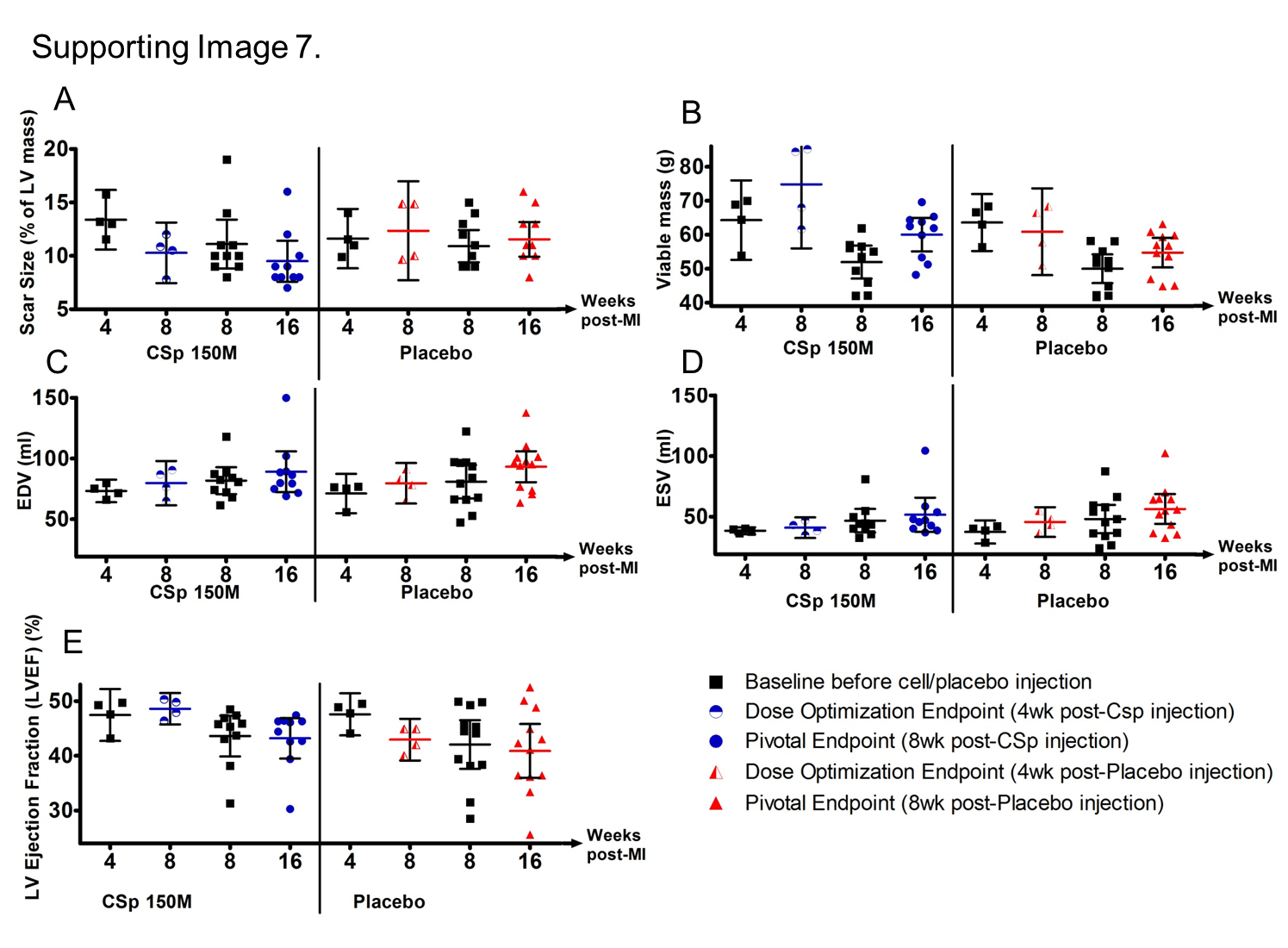

Supplement: Supporting Image S7 — Individual animal MRI data from both dose optimization and pivotal studies, plotted together. Error bars show 95% confidence interval. Time points are given as number of weeks post-MI. A) Scar size decreases with cell injection and increases with placebo in both the dose optimization and pivotal studies. B) Viable left ventricular mass increases with cell injection and decreases with placebo in the dose optimization study. Viable left ventricular mass increases with both cell injection and placebo in the pivotal study, though the increase is less dramatic with placebo. C) End diastolic volume increases with both cell injection and placebo. In the pivotal study, endpoint EDV increases significantly with placebo only, while EDV increases non-significantly with cell injection, corresponding with preservation of left ventricular remodeling. D) End systolic volume increases with both cell injection and placebo. In both the dose optimization and pivotal studies, endpoint ESV increases significantly in placebo while cell injection maintains ESV, corresponding with preservation of left ventricular remodeling. E) Left ventricular ejection fraction decreases with placebo in both the dose optimization and pivotal studies; the decrease is significant in the dose optimization study. Ejection fraction increases non-significantly with cell injection in the dose optimization study and decreases non-significantly with cell injection in the pivotal study. (TIF) [file pone.0113805.s007.tif]
